# De novo assembling and primary analysis of genome and transcriptome of gray whale *Eschrichtius robustus*

**DOI:** 10.1186/s12862-017-1103-z

**Published:** 2017-12-28

**Authors:** Alexey А. Moskalev, Anna V. Kudryavtseva, Alexander S. Graphodatsky, Violetta R. Beklemisheva, Natalya A. Serdyukova, Konstantin V. Krutovsky, Vadim V. Sharov, Ivan V. Kulakovskiy, Andrey S. Lando, Artem S. Kasianov, Dmitry A. Kuzmin, Yuliya A. Putintseva, Sergey I. Feranchuk, Mikhail V. Shaposhnikov, Vadim E. Fraifeld, Dmitri Toren, Anastasia V. Snezhkina, Vasily V. Sitnik

**Affiliations:** 1 Engelhardt Institute of Molecular Biology, Russian Academy of Sciences, Moscow, 119991 Russian Federation; 2 Institute of Biology of Komi Science Center of Ural Branch of RAS, Syktyvkar, 167982 Russian Federation; 3 Institute of Molecular and Cellular Biology SB RAS, Novosibirsk, 630090 Russian Federation; 4 Novosibirsk State University, Novosibirsk, 630090 Russian Federation; 5 Department of Forest Genetics and Forest Tree Breeding, Georg-August University of Göttingen, Göttingen, 37077 Germany; 6 Vavilov Institute of General Genetics, Russian Academy of Sciences, Moscow, 119991 Russian Federation; 7 Genome Research and Education Center, Siberian Federal University, Krasnoyarsk, 660036 Russian Federation; 8 Department of Ecosystem Science and Management, Texas A&M University, College Station, 77843-2138 TX USA; 9 Department of High Performance Computing, Institute of Space and Information Technologies, Siberian Federal University, Krasnoyarsk, 660074 Russian Federation; 10 Center for Data-Intensive Biomedicine and Biotechnology, Skolkovo Institute of Science and Technology, Moscow, 143026 Russia; 11 Irkutsk National Research Technical University, Irkutsk, 664074 Russian Federation; 12 Limnological Institute, Siberian Branch of Russian Academy of Sciences, Irkutsk, 664033 Russian Federation; 13 The Shraga Segal Department of Microbiology, Immunology and Genetics, Faculty of Health Sciences, Center for Multidisciplinary Research on Aging, Ben-Gurion University of the Negev, Beer-Sheva, 8410501 Israel

**Keywords:** Gray whale, *Eschrichtius robustus*, Genome, Transcriptome, DNA repair, Hypoxia-response

## Abstract

**Background:**

Gray whale, *Eschrichtius robustus* (*E. robustus*), is a single member of the family Eschrichtiidae, which is considered to be the most primitive in the class Cetacea. Gray whale is often described as a “living fossil”. It is adapted to extreme marine conditions and has a high life expectancy (77 years). The assembly of a gray whale genome and transcriptome will allow to carry out further studies of whale evolution, longevity, and resistance to extreme environment.

**Results:**

In this work, we report the first de novo assembly and primary analysis of the *E. robustus* genome and transcriptome based on kidney and liver samples. The presented draft genome assembly is complete by 55% in terms of a total genome length, but only by 24% in terms of the BUSCO complete gene groups, although 10,895 genes were identified. Transcriptome annotation and comparison with other whale species revealed robust expression of DNA repair and hypoxia-response genes, which is expected for whales.

**Conclusions:**

This preliminary study of the gray whale genome and transcriptome provides new data to better understand the whale evolution and the mechanisms of their adaptation to the hypoxic conditions.

**Electronic supplementary material:**

The online version of this article (doi: 10.1186/s12862-017-1103-z) contains supplementary material, which is available to authorized users.

## Background

The living marine mammals include five groups: sea otters, polar bears, pinnipeds (seals, sea lions, fur seals, and walruses), sirenians (dugongs and manatees), and cetaceans (whales, dolphins, and porpoises) [1]. The genomic analyses of these animals reveal insights into molecular adaptation to living conditions. For example, the analysis of the polar bear (*Ursus maritimus*) genome revealed a positive selection for genes involved in synthesis of nitric oxide, which can regulate energy production [2]. Comparative genomic analysis of four marine mammalian species, including the walrus (*Odobenus rosmarus*), bottlenose dolphin (*Tursiops truncatus*), killer whale (*Orcinus orca*), and manatee (*Trichechus manatus latirostris*), showed convergent amino acid substitutions in genes evolving under positive selection and putatively associated with a marine phenotype [3]. These genes are linked to changes in bone density (*S100a9*, *Mgp*), formation of the auditory bulla (*Smpx*), the unusual periodic thyroid activity (*C7orf62*), cardiovascular regulation during diving (*Myh7b*), and the low flow rate of viscous blood during diving behavior (*Serpinc1*) [3]. Species-specific evolution of α-keratin gene family identified in the marine mammals, including seven cetaceans, two pinnipeds, polar bear, and manatee might be responsible for their different hair characteristics [4].

The analysis of the minke whale (*Balaenoptera acutorostrata*) genome indicated the signatures of positive selection for genes associated with epilation and tooth-development, supporting the morphological uniqueness of whales [5]. Comparative genomic analysis of the minke whales (*Balaenoptera acutorostrata* and *Balaenoptera bonaerensis*), a fin whale (*Balaenoptera physalus*), a bottlenose dolphin (*Tursiops truncatus*) and a finless porpoise (*Neophocaena phocaenoides*) identified an expansion of genes associated with stress-responsive proteins and anaerobic metabolism, whereas gene families related to body hair and sensory receptors were contracted [6]. Also, the mutations in genes encoding antioxidants and enzymes controlling blood pressure and salt concentration were identified [6]. These features are associated with the physiological and morphological adaptations for life in an aquatic environment, accompanied by a lack of oxygen and high salt levels [6]. The analysis of the genome of bowhead whale (*Balaena mysticetus*), the longest-lived mammal known thus far (over 200 years), identified mutations in genes linked to cancer and aging [7]. In addition, gene gain and loss involving genes associated with DNA repair, cell-cycle regulation, cancer, and aging were identified [7]. The genome-wide gene expression analyses of the *Balaena mysticetus* revealed cetacean-specific changes associated with altered insulin signaling and adaptation to a lipid-rich diet [8].

Gray whale, *Eschrichtius robustus* is a single member of the familyEschrichtiidae. It is one of the four families in the suborder Mysticeti (with the Balaenidae, Neobalaenidae and Balaenopteridae) and is considered to be the most primitive among these families. Gray whale has been described as a “living fossil” because of its short, coarse baleen plates and lack of a dorsal fin [9]. *E. robustus* reaches a length of 14.9 m, a weight of 36 t [10], and lives up to 77 years [11]. The gray whale is distributed throughout coastal areas in the North Pacific. Two gray whale populations are currently recognized: the Western North Pacific population, comprising ~140 individuals, and the Eastern North Pacific (ENP) population, comprising ~20,000 individuals [12]. At the end of the feeding season, the ENP gray whales undertake an 8000-km migration (16,000 km round trip) southward to their winter breeding grounds [12]. They have a breath holding ability. For example, the maximum recorded dive duration for a gray whale clocked in San Ignacio Lagoon was 25.9 min [13]. Chromosomal peculiarities of gray whale and these specimens, including the whole ZooFISH data with human and camel chromosomal painting probes, description and localization of repeated and satellite DNAs were previously reported [14].

Here, we present for the first time de novo assembling, annotation and primary analysis of the *E. robustus* genome and transcriptome of kidney and liver. This study will help to better understand the whale evolution, mechanisms of longevity and adaptation to the life in extreme hypoxic environment.

## Methods

### Animal sample collection

The gray whales used in this study were caught by hunters of the indigenous population of Chukotka Autonomous Okrug (Mechigmen bay of the Bering Sea, Lorino), who have permission to hunt this species for food. Tissue biopsies were taken at the time of aboriginal hunting; no animals were killed specifically for this study.

### Nucleic acid extraction

Genomic DNA was isolated using phenol-chloroform extraction by standard molecular biology techniques. dsDNA was quantified on the Qubit 2.0 Fluorometer (Thermo Fisher Scientific, USA) with the Qubit Broad Range dsDNA kit (Thermo Fisher Scientific, USA), and DNA quality was assessed by electrophoresis in 0.6% agarose gel. Only high-quality DNA with fragments longer than 50 kb was used for the sequencing library preparation.

Total RNA was isolated from liver and kidney tissues of the same individual using the RNeasy Mini Kit (QIAGEN, Germany) according to the manufacturer’s protocol. RNA quantification was performed on the NanoDrop 1000 (NanoDrop Technologies, USA), and the RNA integrity was assessed using the Agilent 2100 Bioanalyzer (Agilent Technologies, USA). RNA was further threated with DNase I (Thermo Fisher Scientific, USA) and purified using the RNA Clean & Concentrator-5 kit (Zymo Research, USA).

### Whole genome sequencing

Three genomic DNA libraries were constructed according to the Illumina recommendations - two mate-pair (MP) libraries from 5 Kb and 10 Kb long fragment sizes using the Nextera Mate Pair Library Prep Kit (Illumina, USA) and one paired-end (PE) library with an insert average size of ~300 bp using the TruSeq DNA Library Prep Kit LT (Illumina, USA) according to the manufacturer’s recommendations. The whole genome sequencing was performed by the Genotek company (Moscow, Russia) on the Illumina HiSeq 2500 with 2 × 75 bp PE and 2 × 100 bp MP sequencing.

### Transcriptome sequencing

The cDNA libraries were prepared using the Illumina TruSeq RNA Sample Preparation Kit v2 (LT protocol) as described in [15]. The libraries were sequenced on the Illumina MiSeq System (USA) using the MiSeq Reagent Kit v2 for 500 (2 × 250) cycles. The sequencing was carried out in the Genome Center of V.A. Engelhardt Institute of Molecular Biology of the Russian Academy of Sciences (EIMB RAS, Moscow, Russia). Statistics of sequencing for transcriptome data are presented in the Table 1.

**Table 1:** The gray whale transcriptome sequencing statistics

Sample	Reads length (Illumina PE), bp	Number of reads (pairs)
Kidney	250 × 2	13,785,570
Liver	250 × 2	22,442,394

### Genome assembly

The software package CLC Assembly Cell (QIAGEN Bioinformatics, USA) was used for genome assembly using sequencing reads generated from all three libraries (Table 2). The main summary statistics of the genome assembly is presented in Table 3.

**Table 2:** Libraries sequenced for the gray whale genome assembly

Illumia library	Reads length, bp	Number of reads (pairs)
PE with a 300 bp insert	75 × 2	39,011,360
MP from 5 Kb fragments	100 × 2	200,299,976
MP from 10 Kb fragments	100 × 2	175,370,211

**Table 3:** Main summary statistics of the final gray whale genome assembly

Assemply	Total number	N50, Kb	Longest, Kb	Total length, Gb
Contigs	1,595,257	2.66	45.5	2.008
Scaffolds	1,213,011	10.67	152.01	2.923 (~31% Ns)

The sequencing reads were trimmed to remove adapters and low quality reads using the trimmomatic v. 0.36 software with the following parameters: the minimum read length and quality were set to 40 bp and 23 (phred-score) in the window size of 4 bp, respectively. After removing 5,929,633 reads (~15.2%) from the original raw 39,011,360 PE reads 30,804,982 × 2 (61,609,964) PE reads and 2,276,745 single end reads (i.e., the PE reads that lost their pair) were used further for assembling. Similarly, after removing 13,251,061 (~6.6%) and 6,627,724 (~3.8%) reads from the original raw 200,299,976 × 2 and 175,370,211 × 2 MP reads representing 5 Kb and 10 Kb fragments, respectively, 119,193,555 × 2 (238,387,110) MP reads and 2,276,745 single end reads (i.e., the MP reads that lost their pair) for the 5 Kb MP library and 113,663,072 × 2 (227,326,144) MP reads and 113,663,072 single end reads for the 10 Kb MP library were used further for scaffolding. Finally, 597,389,628 reads with a total length of 53,174,027,264 bp (16.6× coverage) were assembled using the clc_assembler in the CLC Assembly Cell v. 4.4.2. software package with the word_size parameter equaled 26. Scaffolding was done automatically as one of the steps while executing the clc_assembler program.

### Basic genome annotation

The annotation was carried out using a set of software packages and databases (Additional file 1). The primary model for marking the position of genes was obtained by the BUSCO package [16] (Additional file 2). A subset of 3023 groups for Vertebrata was considered. For the detection of genes the AUGUSTUS package [17] with the initial model “human” (*H. sapiens*) was used (Additional file 3). The masking was performed with the RepeatMasker package [18] using the RepBase repeats libraries [19] and Dfam [20]. Annotation was carried out with scripts based on the funannotate pipeline [21].

The protein and transcriptomic hints for marking the position of genes were also used. Protein hints were obtained using the Exonerate package [22] (with the appropriate funannotate wrapper) and the protein sequences database SwissProt [23] (for Vertebrata) as well as the protein sequences from the minke whale and bowhead whale assemblies (Additional file 2). Transcriptomic hints were obtained using the BLAT tool [24] with the provided transcriptome assembly. The primary locations of genes obtained using AUGUSTUS was reformatted using the EVidence Modeller package [25] (with the appropriate funannotate wrapper). The finalization of the primary position of genes was carried out using the funannotate pipeline. In total, the primary annotation found 152,339 exons from 43,456 parts of genes.

### Functional annotation of the genome

Search for tRNA genes in genomic sequence was performed with tRNAscan-SE program [26]. The predicted variants with score above 65, not pseudo, and not undetermined were selected to the final annotation. As a result, the final annotation included 259 predicted tRNAs.

Functional annotation was started by the funnannotate pipeline with disabled annotation by InterPro resource [27, 28]. An annotation was made with the SwissProt protein sequence database [23], Pfam protein families database [29], eggNOG database [30], MEROPS peptidase database [31], and BUSCO families [16]. If protein sequence for the gene was not found in SwissProt, a search for homologs among model mammals in the NCBI Landmark database was conducted.

Then, the filtering stage of the marked genes followed. At this stage, only genes with clarified descriptions in SwissProt/NCBI Landmark were selected. One top hit was considered for each marked gene. The total number of unfiltered fragments was 28,260, unique hits – 18,261, with one hit – 12,411. On the average, the one hit had 1.5 gene fragments, and fragmented genes were divided into 2.7 parts. The tRNA genes were not filtered.

At filtering stage found genes were selected when more than 30% of the hit from the database were covered by the gene with identity above 60%, and the hit from the database covered more than 60% of the gene. If several genes were found from the database in the same hit, the longest variant was selected. If the top hits for different parts had different IDs (homologues from different organisms), this approach admits annotation of different parts of the same gene, as different genes. Unfortunately, this approach is strongly biased, reduces completeness, does not allow to reveal duplications, but allowed to follow some limitations on the number and quality of gene marking. After filtering, funannotate pipeline was started again with the annotation by InterPro and GO terms (Table 4; Additional file 4).

**Table 4:** Main summary statistics of the genome functional annotation

Genome elements	Number	Percentage of the whole 2.9 Gb assembly
Repeats	3,473,947	22.96%
Genes (not including tRNA)	10,894	2.29% (0.38% for CDS)
Exons	56,837	0.3579%
tRNA	259	0.0007%

### Phylogenetic analysis

Phylogenetic trees were constructed based on multiple alignments for 322 groups of single-copy orthologous genes found by the BUSCO methodology for 16 organisms obtained from the NCBI and Ensembl repositories [32] (Additional file 5). The corresponding protein sequences and CDS for 5152 genes were aligned.

The search for single-copy orthologs was carried out using BUSCO [16]. For the genes represented by several transcripts, only one transcript (with protein product) was selected with the largest BUSCO score. The genes that have one copy in all considered genomes (“complete”, in terms of BUSCO) were selected for analysis.

The CDS corresponding to the selected 322 gene groups was aligned using the MAFFT program [33] in the E-INS-i mode, focused on the quality of alignment (with the parameters --ep 0 --genafpair - maxiterate 2000). The resulting alignments were processed by the GBlocks program [34] and concatenated together into one long sequence. The total length of the sequences for the phylogenetic analysis for CDS was 252,271 base pairs.

The consensus phylogenetic tree was constructed using the RAxML software [35] with the GTRGAMMAI model. To estimate the convergence of the bootstrapping the autoMRE criterion (extended majority rule consensus tree criterion) was used. The tree of species divergence was constructed by the BEAST package [36] with the HKY + Gamma model. The a priori restrictions on divergence times [37] are given in Additional file 6.

### De novo transcriptome assembly

The RNA-Seq reads of liver and kidney samples were pooled, trimmed with Trimmomatic [38] (with default recommended parameters except for SLIDINGWINDOW:4:20 MINLEN:36), pooled and supplied to Trinity [39] to perform de novo transcriptome assembly. The resulting transcriptome assembly from the four pooled samples contained 114,233 contigs.

### Comparison of transcriptome assemblies

In our comparative analysis, we used the published whale transcriptome and genome data [6–8]. The details are provided in the Additional file 7. To map transcriptome contigs against bowhead whale genome CDS (which is more complete than our assembly) and Alaska bowhead whale transcriptome, we used the best hits of blast (executed with default parameters) [40].

### Annotation of the obtained gray whale transcriptome assembly and differential gene expression analysis

We used TransDecoder to predict ORFs in assembled contigs and Trinotate [39, 41] to annotate ORFs based on similarity to known orthologous genes. The complete resulting annotation is provided in the Additional file 8, the predicted ORFs are included as an Additional file 9.

To assess gene expression we mapped transcriptome reads of several whale transcriptomes using the gray whale transcriptome assembly as a reference. The reads were trimmed with sickle [42] and cutadapt [43] and mapped using bowtie2 [44] to all contigs carrying ORFs predictions. Usage of a non-conspecific reference may require special optimization of mapping parameters, but in our case, the mapping success rate was rather high for all used transcriptomes. The use of annotated genome could be more relevant but is of limited value due to the overall incompleteness of the produced genome assembly [45].

The mappings in unpaired mode were quite good with nearly 90% of the gray whale reads successfully mapped (80% for minke whale and bowhead whale reads). The mapping in paired mode showed lower but reasonable success rate (70% for gray whale and more than 50% for bowhead and minke whale data). The unpaired mappings were then used for read counting and gene expression analysis to reduce loss of information. The overall statistics are given in the Additional file 10.

The read counting was performed with HTSeq [46]. Complete read counts are given in the Additional file 11, the distribution of read counts per contig is provided in Additional file 12. Differential expression was assessed with edgeR [47]. One count-per-million expression threshold was used to select the set of reliably expressed transcripts. Only 10% of chimeric contigs (with two or more predicted ORFs) passed this expression threshold, which supports the reliability of the transcriptome assembly and annotation. The GO enrichment analysis was performed with the Fisher’s exact test.

## Results and discussion

### Draft whole genome sequence assembly and annotation

A whole-genome shotgun sequence approach was used to the genome assembly of the gray whale (*E. robustus*). The liver and kidney transcriptomes were also sequenced and assembled. Approximately 53 Gb (16.6× coverage assuming 3.19 (±0.5) Gb of an average Cetacean genome size [48]) genome data were generated. The Illumina PE and two MP libraries were sequenced, and obtained reads were used for genome assembly (Table 2). The draft assembly was built with the CLC Assembly Cell (QIAGEN Bioinformatics, USA) software package. Due to additional filtration during genome submission to the NCBI Genbank database many scaffolds were removed leaving finally 1,213,011 scaffolds with N50 of 10.67 Kb (Table 3).

The data of the transcriptome assembly were used for the genome annotation. The primary assessment of genome assembly was carried out using the BUSCO methodology [16]. The number, fragmentation and duplication level of unique orthologs from the different species were evaluated. The genome assemblies of minke whale (*Balaenoptera acutorostrata scammoni*), bowhead whale (*Balaena mysticetus*), and Antarctic minke whale (*Balaenoptera bonaerensis*) were used for comparison (Additional files 2 and 3).

Based on the primary BUSCO analysis, the expected number of completely reconstituted genes (including duplicated) was 24%. Apparently, this is due to the relatively small N50 for scaffolds and contigs, although comparable with the median length for genes in related species (for instance, ~ 9.3 Kb for minke whale) (Table 3; Additional file 3).

Known repeats and sequences with low complexity comprised about 22.96% of the entire assembly (671.01 Mb) (Table 4; Methods). Despite the fragmented assembly (152,339 exons from 43,456 parts of genes were initially found), the selection of the contigs with the longest gene fragments (see Methods) allowed to mark 10,894 genes (56,837 exons) (Table 4; Additional file 4).

### Phylogenetic analysis

**Fig. 1:**
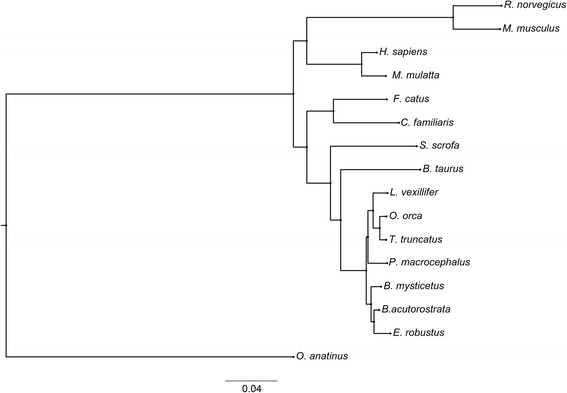
Phylogenetic tree based on 322 groups of the CDS sequences of the single-copy orthologous genes. The length of the edges is proportional to the number of substitutions per site. The bootstrap value for all nodes was 100

**Fig. 2:**
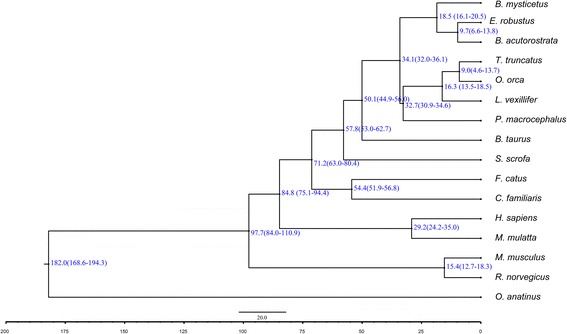
Phylogenetic species tree was based on multiple alignments for CDS. A priori restrictions on divergence times were used (Additional file 6). The values of the discrepancy time and 95% confidence intervals are shown at nodes

Phylogenetic trees were reconstructed based on multiple alignments for 322 groups of single-copy orthologous genes from 16 organisms (Additional file 5). Single-copy “complete” groups were selected in terms of the BUSCO methodology. Figure 1 shows a phylogenetic tree obtained from multiple alignments of examined groups of protein sequences. Despite the insignificant completeness of the genome in terms of genes (about 24% complete based on the BUSCO estimate, see Additional file 3), the used approach allowed the construction of a plausible tree for groups of protein sequences, keeping the dense of Cetacea cluster. Figure 2 shows a tree of species divergence obtained by multiple alignments of CDS. The used a priori limitations on divergence times [37] are given in the Additional file 6. Unfortunately, because of the incompleteness of the draft assembly, there are some deviations in the estimates of the species divergence time from the median estimates given in the TimeTree resource [37]. At the same time, the estimated divergence time of *O. orca* and *E. robustus* (34.1, CI: (32.0–36.1) MYA) slightly differs from the median time (34.4 CI: (30.6–35.5 MYA)) given on the same resource.

### The produced gray whale transcriptome assembly provides a better representation of the whale transcriptome compared to previously published data

The genome assembly produced in our study is of notably lower reliability than the complementary transcriptome assembly**.** For comparison, the other published bowhead whale transcriptome assemblies are less realistic with 423,657 and 1,059,024 contigs, respectively [7]. Thus, for comparative analysis we additionally utilized the genome CDS annotation (22,677 CDSs) of the bowhead whale [7] (Table 5).

**Table 5:** Comparative data on the whale transcriptome assemblies

	Gray whale	Bowhead whale (Alaska)	Bowhead whale (Greenland)	Bowhead whale (CDS)
Number of contigs	114,233	423,657	1,059,024	22,677
Total length of contigs	79,386,154	401,340,157	754,726,832	28,384,452
N50	1280	2436	1283	1671

In fact, the total number of contigs of the gray whale transcriptome assembly is ten times smaller than of the other existing transcriptomes, and its N50 value is reasonably close to that of the bowhead whale genomics CDSs. This suggests that the produced transcriptome assembly has less ‘false positive’ and redundant contigs than other published assemblies. To support this statement, we mapped all tested transcriptomes against bowhead whale genome CDS, as well as Greenland bowhead whale and gray whale transcriptomes against the middle-sized Alaska bowhead whale transcriptome. In both tests the mapping showed 2–10 times higher fraction of mapped contigs for the gray whale transcriptome (Additional file 7). Furthermore, the absolute number of reliably mapped contigs and genome CDSs covered by mapped transcriptome contigs were similar for all three tested transcriptome assemblies, which is surprising giving dramatically smaller total size of the gray whale transcriptome assembly. Inter-transcriptome mapping also supports this observation (Additional file 10).

### Consistent gene expression across different whale transcriptome samples supports reliability of the transcriptome assembly and annotation

To comparatively assess gene expression profiles in kidney and liver of the gray whale we performed standard gene expression analysis using the de novo assembled transcriptome as the reference. The kidney is involved in regulation of the water balance, volume and composition of the blood [49, 50]. The liver is critical in digestive function and metabolism, production of various plasma proteins, immune function, and detoxification of xenobiotics [51, 52]. The central roles of the kidney and liver in many aspects of whole-body physiology makes the hepatic and renal transcriptomes pivotal for understanding normal homeostasis and mechanisms of adaptation to the conditions of existence. The gene expression patterns in the same organs of different whale species were very similar exhibiting only a limited number of differentially expressed genes. In particular, we detected robust expression of DNA repair and hypoxia-response genes. Genetic instability and chronic tissue hypoxia are the main mechanisms related to both aging and longevity [53, 54]. It is known that the long-lived species have a high level of DNA repair genes activity and are resistant to unfavorable environmental conditions [7, 55]. Marine mammals should have an increased resistance to hypoxia due to their breath holding ability [6, 56]. Thus, robust expression of the DNA repair and hypoxia-response genes may reflect the adaptation of the gray whale to the life in hypoxic environment and may, to some extent, explain its longevity. All in all, this is the first proof of the possible involvement of hypoxia-response genes in longevity determination in whales.

Next, we performed the gene ontology (GO) enrichment analysis for genes exhibiting significantly higher expression in the gray whale transcriptome (against minke and bowhead whale data). Technically, we used the gray whale transcriptome assembly as the reference to map RNA-Seq reads from other whale transcriptomes, and this could reduce the power of the differential expression analysis. Indeed, there were almost no differential expression detected for kidney samples, and the GO analysis did not show any relevant enrichment. GO enrichment analysis of liver data found multiple GO terms enriched (see Additional files 12 and 13), which are mostly linked to the xenobiotic stress response.

## Conclusions

We made de novo assembling and primary analysis of gray whale (*E. robustus*) genome and transcriptome of kidney and liver. According to the estimation by the BUSCO methodology, the completeness of the draft genome assembly was about 24%. After selecting the longest contigs, 10,894 genes were found. The repeats represented about 22.96% of the entire assembly. The transcriptome analysis revealed robust expression of DNA repair and hypoxia-response genes, which is consistent with the adaptation of whales to deep diving. The GO enrichment analysis demonstrated increased expression of genes related to xenobiotic stress response in the gray whale liver. This can be due to both the habitat conditions and the physiological state of the individual. Further study of the genome and transcriptome of the gray whale may be useful for understanding the evolution of whales, mechanisms of longevity and adaptation to hypoxic conditions.

## Additional files

Additional file 1: The versions of used software packages and databases. (PDF 127 kb)

Additional file 2: Assemblies for primary comparison with BUSCO. (PDF 110 kb)

Additional file 3: The primary analysis with the BUSCO methodology. (PDF 111 kb)

Additional file 4: Functional annotation of genes with funannotate. (PDF 8 kb)

Additional file 5: Genomic data used for phylogenetic analysis. (PDF 29 kb)

Additional file 6: A priori estimates of the dates of divergence obtained by using TimeTree resource. (PDF 110 kb)

Additional file 7: Comparative assessment of the resulting gray whale transcriptome assembly. (PDF 127 kb)

Additional file 8: The complete resulting annotation of the gray whale transcriptome assembly. (XLSX 13480 kb)

Additional file 9: The predicted ORFs. (XLSX 10574 kb)

Additional file 10: Transcriptome read mapping statistics. See Additional file 7 for the data sources overview. (PDF 12 kb)

Additional file 11: Complete read counts. (XLSX 7180 kb)

Additional file 12: Differential gene expression. (PDF 377 kb)

Additional file 13: GO analysis. (XLSX 9 kb)

## Abbreviations

bp: Base pairs
CDS: Coding DNA sequence
Gb: Gigabase pairs
GO: Gene ontology
Kb: Kilobase pairs
Mb: Megabase pairs
MYA: Million years ago
ORF: Open reading frame
tRNA: Transfer RNA
