# Supplementary material for: De novo assembling and primary analysis of genome and transcriptome of gray whale Eschrichtius robustus
Source: BMC Evol Biol. 2017 Dec 28;17(Suppl 2):258. doi: 10.1186/s12862-017-1103-z (PMC5751776; doi:10.1186/s12862-017-1103-z)
Supplement: Supplementary file 1 — The versions of used software packages and databases. (PDF 127 kb) [file 12862_2017_1103_MOESM1_ESM.pdf]

The versions of used software packages and databases

Package/Base	Version/Date of circulation
funannotate [1]	0.3.4
tRNAscan [2]	1.4
BUSCO [3]	1.22
Exonerate [4]	2.2.0
AUGUSTUS [5]	3.2.2
EVidenceModeller [6]	0.1.3
MAFFT [7]	7.305
RAxML [8]	8.2.9
GBlocks [9]	0.91b
RepeatMasker [10]	4.0.6
BEAST [11]	2.4.4
Blat [12]	36
InterPro [13]	58.0, Aug 17, 2016
InterProScan [14]	version 5.19-58.0, accessed with rest
maNOG [15]	4.5
Pfam [16]	Aug 17, 2016
MEROPS [13]	Aug 2, 2016
Repbase [17]	20150807
Dfam [18]	20150807

References

1. Palmer JM. Funannotate: pipeline for genome annotation. http://www.github.com/nextgenusfs/funannotate. 2016.
2. Lowe TM, Eddy SR. tRNAscan-SE: a program for improved detection of transfer RNA genes in genomic sequence. Nucleic Acids Res. 1997;25:955-64.
3. Simão FA, Waterhouse RM, Ioannidis P, Kriventseva EV, Zdobnov EM. BUSCO: assessing genome assembly and annotation completeness with single-copy orthologs. Bioinformatics. 2015;31:3210-12.
4. Slater GS, Birney E. Automated generation of heuristics for biological sequence comparison. BMC Bioinformatics. 2005;6:31.
5. Keller O, Kollmar M, Stanke M, Waack S. A novel hybrid gene prediction method employing protein multiple sequence alignments. Bioinformatics. 2011;27:757-63.
6. Haas BJ, Salzberg SL, Zhu W, Pertea M, Allen JE, Orvis J, White O, Buell CR, Wortman JR. Automated eukaryotic gene structure annotation using EVidenceModeler and the Program to Assemble Spliced Alignments. Genome Biol. 2008;9:R7.
7. Katoh K, Standley DM. A simple method to control over-alignment in the MAFFT multiple sequence alignment program. Bioinformatics. 2016;32:1933-42.
8. Stamatakis A. RAxML version 8: a tool for phylogenetic analysis and post-analysis of large phylogenies. Bioinformatics. 2014;30:1312-3.
9. Talavera G, Castresana J. Improvement of phylogenies after removing divergent and ambiguously aligned blocks from protein sequence alignments. Syst Biol. 2007;56:564-77.
10. Smit AFA, Hubley R, Green P. RepeatMasker Open-4.0. 2013-2015 http://www.repeatmasker.org.
11. Drummond AJ, Suchard MA, Xie D, Rambaut A. Bayesian phylogenetics with BEAUti and the BEAST 1.7. Molecular biology and evolution. 2012;29:1969-73.
12. Kent WJ. BLAT — the BLAST-like alignment tool. Genome Res. 2002;12:656-64.
13. Rawlings ND, Barrett AJ, Bateman A. MEROPS: the database of proteolytic enzymes, their substrates and inhibitors. Nucleic Acids Res. 2012;40:D343-50.
14. Sangrador-Vegas A, Mitchell AL, Chang HY, Yong SY, Finn RD. GO annotation in InterPro: why stability does not indicate accuracy in a sea of changing annotations. Database (Oxford). 2016;2016.
15. Huerta-Cepas J, Szklarczyk D, Forslund K, Cook H, Heller D, Walter MC, Rattei T, Mende DR, Sunagawa S, Kuhn M, Jensen LJ, von Mering C, Bork P. eggNOG 4.5: a hierarchical orthology framework with improved functional annotations for eukaryotic, prokaryotic and viral sequences. Nucleic Acids Res. 2016;44:D286-93.
16. Finn RD, Coggill P, Eberhardt RY, Eddy SR, Mistry J, Mitchell AL, Potter SC, Punta M, Qureshi M, Sangrador-Vegas A, Salazar GA, Tate J, Bateman A. The Pfam protein families database: towards a more sustainable future. Nucleic Acids Res. 2016;44:D279-85.
17. Bao W, Kojima KK, Kohany O. Repbase Update, a database of repetitive elements in eukaryotic genomes. Mobile DNA. 2015;6:11.
18. Hubley R, Finn RD, Clements J, Eddy SR, Jones TA, Bao W, Smit AF, Wheeler TJ. The Dfam database of repetitive DNA families. Nucleic Acids Res. 2016;44:D81-9.
