# Supplementary material for: De novo assembling and primary analysis of genome and transcriptome of gray whale Eschrichtius robustus
Source: BMC Evol Biol. 2017 Dec 28;17(Suppl 2):258. doi: 10.1186/s12862-017-1103-z (PMC5751776; doi:10.1186/s12862-017-1103-z)
Supplement: Supplementary file 2 — Assemblies for primary comparison with BUSCO. (PDF 110 kb) [file 12862_2017_1103_MOESM2_ESM.pdf]

Assemblies for primary comparison with BUSCO [1]

Name	Sources	N50, Kb
Minke whale (*Balaenoptera acutorostrata scammoni*)	http://www.ncbi.nlm.nih.gov/assembly/GCF_000493695.1/	Scaffold N50 12,843.7 Kb<br>Contig N50 22.7 Kb
Bowhead whale (*Balaena mysticetus*)	http://alfred.liv.ac.uk/downloads/bowhead_whale/bowhead_whale_proteins.zip<br>http://alfred.liv.ac.uk/downloads/bowhead_whale/bowhead_whale_scaffolds.zip	Scaffolds N50 877 Kb<br>Contigs N50 34.8 Kb
Antarctic minke whale (*Balaenoptera bonaerensis*)	http://www.ncbi.nlm.nih.gov/assembly/GCA_000978805.1	Scaffold N50 20 Kb<br>Contig N50 8 Kb

References

1. Simão FA, Waterhouse RM, Ioannidis P, Kriventseva EV, Zdobnov EM. BUSCO: assessing genome assembly and annotation completeness with single-copy orthologs. Bioinformatics. 2015;31:3210-12.
