# Supplementary material for: De novo assembling and primary analysis of genome and transcriptome of gray whale Eschrichtius robustus
Source: BMC Evol Biol. 2017 Dec 28;17(Suppl 2):258. doi: 10.1186/s12862-017-1103-z (PMC5751776; doi:10.1186/s12862-017-1103-z)
Supplement: Supplementary file 3 — The primary analysis with the BUSCO methodology. (PDF 111 kb) [file 12862_2017_1103_MOESM3_ESM.pdf]

The primary analysis with the BUSCO methodology [1]

Source	Complete BUSCOs (%)	Duplicated, (included in complete) (%)	Fragmented (%)	Missing, %
Grey whale/initial	24	0.4	24	50
Minke whale/proteins	97	44	1.5	0.6
Bowhead whale/genome	60	1.3	25	13
Bowhead whale/proteins	72	2	14	12
Antarctic minke whale	NA	NA	NA	NA

References

1. Simão FA, Waterhouse RM, Ioannidis P, Kriventseva EV, Zdobnov EM. BUSCO: assessing genome assembly and annotation completeness with single-copy orthologs. Bioinformatics. 2015;31:3210-12.
