# Supplementary material for: De novo assembling and primary analysis of genome and transcriptome of gray whale Eschrichtius robustus
Source: BMC Evol Biol. 2017 Dec 28;17(Suppl 2):258. doi: 10.1186/s12862-017-1103-z (PMC5751776; doi:10.1186/s12862-017-1103-z)
Supplement: Supplementary file 4 — Functional annotation of genes with funannotate. (PDF 8 kb) [file 12862_2017_1103_MOESM4_ESM.pdf]

Functional annotation of genes with funannotate [1]

Total genes (with description)	10895
EggNog	10032
InterPro	9995
GO	7704
BUSCO	736
PFAM	1835
MEROPS	452

References

1. Palmer JM. Funannotate: pipeline for genome annotation. http://www.github.com/nextgenusfs/funannotate. 2016.
