# Supplementary material for: De novo assembling and primary analysis of genome and transcriptome of gray whale Eschrichtius robustus
Source: BMC Evol Biol. 2017 Dec 28;17(Suppl 2):258. doi: 10.1186/s12862-017-1103-z (PMC5751776; doi:10.1186/s12862-017-1103-z)
Supplement: Supplementary file 5 — Genomic data used for phylogenetic analysis. (PDF 29 kb) [file 12862_2017_1103_MOESM5_ESM.pdf]

Genomic data used for phylogenetic analysis

Used name	Species	Source	Link to proteins	Link to CDS
Human	*H. sapiens*	ensembl release-87	ftp://ftp.ensembl.org/pub/release-87/fasta/homo_sapiens/pep/Homo_sapiens.GRCh38.pep.all.fa.gz	ftp://ftp.ensembl.org/pub/release-87/fasta/homo_sapiens/cds/Homo_sapiens.GRCh38.cds.all.fa.gz
Mouse	*M. musculus*	ensembl release-87	ftp://ftp.ensembl.org/pub/release-87/fasta/mus_musculus/pep/Mus_musculus.GRCm38.pep.all.fa.gz	ftp://ftp.ensembl.org/pub/release-87/fasta/mus_musculus/cds/Mus_musculus.GRCm38.cds.all.fa.gz
Rat	*R. norvegicus*	ensembl release-87	ftp://ftp.ensembl.org/pub/release-87/fasta/rattus_norvegicus/pep/Rattus_norvegicus.Rnor_6.0.pep.all.fa.gz	ftp://ftp.ensembl.org/pub/release-87/fasta/rattus_norvegicus/cds/Rattus_norvegicus.Rnor_6.0.cds.all.fa.gz
Cat	*F. catus*	ensembl release-87	ftp://ftp.ensembl.org/pub/release-87/fasta/felis_catus/pep/Felis_catus.Felis_catus_6.2.pep.all.fa.gz	ftp://ftp.ensembl.org/pub/release-87/fasta/felis_catus/cds/Felis_catus.Felis_catus_6.2.cds.all.fa.gz
Cow	*B. taurus*	ensembl release-87	ftp://ftp.ensembl.org/pub/release-87/fasta/bos_taurus/pep/Bos_taurus.UMD3.1.pep.all.fa.gz	ftp://ftp.ensembl.org/pub/release-87/fasta/bos_taurus/cds/Bos_taurus.UMD3.1.cds.all.fa.gz
Dog	*C. familiaris*	ensembl release-87	ftp://ftp.ensembl.org/pub/release-87/fasta/canis_familiaris/pep/Canis_familiaris.CanFam3.1.pep.all.fa.gz	ftp://ftp.ensembl.org/pub/release-87/fasta/canis_familiaris/cds/Canis_familiaris.CanFam3.1.cds.all.fa.gz
Macaque	*M. mulatta*	ensembl release-87	ftp://ftp.ensembl.org/pub/release-87/fasta/macaca_mulatta/pep/Macaca_mulatta.Mmul_8.0.1.pep.all.fa.gz	ftp://ftp.ensembl.org/pub/release-87/fasta/macaca_mulatta/cds/Macaca_mulatta.Mmul_8.0.1.cds.all.fa.gz
Dolphin	*T. truncatus*	ensembl release-87	ftp://ftp.ensembl.org/pub/release-87/fasta/tursiops_truncat	ftp://ftp.ensembl.org/pub/release-87/fasta/tursiops_truncat

			us/pep/Tursiops_truncatus.turTru1.pep.all.fa.gz	us/cds/Tursiops_truncatus.turTru1.cds.all.fa.gz
Pig	*S. scrofa*	ensembl release-87	ftp://ftp.ensembl.org/pub/release-87/fasta/sus_scrofa/pep/Sus_scrofa.Sscrofa10.2.pep.all.fa.gz	ftp://ftp.ensembl.org/pub/release-87/fasta/sus_scrofa/cds/Sus_scrofa.Sscrofa10.2.cds.all.fa.gz
Minke whale	*B. acutorostrata*	NCBI	ftp://ftp.ncbi.nlm.nih.gov/genomes/all/GCF/000/493/695/GCF_000493695.1_BalAcu1.0/GCF_000493695.1_BalAcu1.0_protein.faa.gz	ftp://ftp.ncbi.nlm.nih.gov/genomes/all/GCF/000/493/695/GCF_000493695.1_BalAcu1.0/GCF_000493695.1_BalAcu1.0_cds_from_genomic.fna.gz
Yangzte river dolphin	*L. vexillifer*	NCBI	ftp://ftp.ncbi.nlm.nih.gov/genomes/all/GCF/000/442/215/GCF_000442215.1_Lipotes_vexillifer_v1/GCF_000442215.1_Lipotes_vexillifer_v1_protein.faa.gz	ftp://ftp.ncbi.nlm.nih.gov/genomes/all/GCF/000/442/215/GCF_000442215.1_Lipotes_vexillifer_v1/GCF_000442215.1_Lipotes_vexillifer_v1_cds_from_genomic.fna.gz
Killer whale	*O. orca*	NCBI	ftp://ftp.ncbi.nlm.nih.gov/genomes/all/GCF/000/331/955/GCF_000331955.2_Oorc_1.1/GCF_000331955.2_Oorc_1.1_protein.faa.gz	ftp://ftp.ncbi.nlm.nih.gov/genomes/all/GCF/000/331/955/GCF_000331955.2_Oorc_1.1/GCF_000331955.2_Oorc_1.1_cds_from_genomic.fna.gz
Sperm whale	*P. macrocephalus*	NCBI	ftp://ftp.ncbi.nlm.nih.gov/genomes/all/GCF/000/472/045/GCF_000472045.1_Physeter_macrocephalus-2.0.2/GCF_000472045.1_Physeter_macrocephalus-2.0.2_protein.faa.gz	ftp://ftp.ncbi.nlm.nih.gov/genomes/all/GCF/000/472/045/GCF_000472045.1_Physeter_macrocephalus-2.0.2/GCF_000472045.1_Physeter_macrocephalus-2.0.2_cds_from_genomic.fna.gz
Platypus	*O. anatinus*	ensembl release-87	ftp://ftp.ensembl.org/pub/release-	ftp://ftp.ensembl.org/pub/release-

			87/fasta/ornithorhynchus_anatinus/pep/Ornithorhynchus_anatinus.OANA5.pep.all.fa.gz	87/fasta/ornithorhynchus_anatinus/cds/Ornithorhynchus_anatinus.OANA5.cds.all.fa.gz
Bowhead whale	*B. mysticetus*	http://www.bowhead-whale.org/	http://alfred.liv.ac.uk/downloads/bowhead_whale/bowhead_whale_proteins.zip	http://alfred.liv.ac.uk/downloads/bowhead_whale/bowhead_whale_coding_sequences.zip
