# Supplementary material for: De novo assembling and primary analysis of genome and transcriptome of gray whale Eschrichtius robustus
Source: BMC Evol Biol. 2017 Dec 28;17(Suppl 2):258. doi: 10.1186/s12862-017-1103-z (PMC5751776; doi:10.1186/s12862-017-1103-z)
Supplement: Supplementary file 6 — A priori estimates of the dates of divergence obtained by using TimeTree resource. (PDF 110 kb) [file 12862_2017_1103_MOESM6_ESM.pdf]

A priori estimates of the dates of divergence obtained by using TimeTree resource [1]

Species		Time of divergence (million years ago)	
		Estimated time	CI
Platypus	All	177	164-191
Dog	Cat	54.6	52-57.3
Minke whale	Sperm whale	33.5	30.6–36.7
Human	Macaque	27.7	23.4–32.7
Rat	Mouse	24.6	16.6-36.4
Yangtze river dolphin	Sperm whale	34	31-37.3

References

1. Kumar S, Stecher G, Suleski M, Hedges SB. TimeTree: A Resource for Timelines, Timetrees, and Divergence Times. Molecular biology and evolution. 2017;34:1812-19.
