# Supplementary material for: De novo assembling and primary analysis of genome and transcriptome of gray whale Eschrichtius robustus
Source: BMC Evol Biol. 2017 Dec 28;17(Suppl 2):258. doi: 10.1186/s12862-017-1103-z (PMC5751776; doi:10.1186/s12862-017-1103-z)
Supplement: Supplementary file 7 — Comparative assessment of the resulting gray whale transcriptome assembly. (PDF 127 kb) [file 12862_2017_1103_MOESM7_ESM.pdf]

Comparative assessment of the resulting gray whale transcriptome assembly

Data sources

For general comparison we used bowhead whale transcriptome assemblies published by Keane et al., which correspond to Alaska and Greenland specimens, and the respective genome assembly [1].

For analysis of differential gene expression we used raw data on liver and kidney transcriptomes from bowhead whale [2] and minke whale [3], see also the Table 10 in the Supplementary materials of the last paper.

Comparative assessment based on published transcriptome assemblies

Table S1 General assessment of the transcriptome assemblies

	Gray whale transcriptome	Bowhead whale transcriptome (Alaska)	Bowhead whale transcriptome (Greenland)	Bowhead whale genome (CDS)
Total number of contigs	114233	423657	1059024	22677
N50	1280	2436	1283	1671

It is worth noting, that both bowhead whale transcriptomes have unrealistic total number of contigs, with an extreme value over a million for the Greenland specimen transcriptome. The N50 value is too high for the Alaska specimen. All in all, both bowhead whale transcriptomes seem to contain an increased number of ‘false positive’ contigs.

Table S2 Comparison of the transcriptome assemblies versus the genome assembly CDSs

	Gray whale transcriptome	Bowhead whale transcriptome (Alaska)	Bowhead whale transcriptome (Greenland)
Number of mapped contigs (transcriptome to genome)	35242	84465	131617
Number of CDSs covered by transcriptome contig hits	17880	20457	19557
Number of reliably mapped contigs (>0.7 length and > 0.7 identity for a transcriptome contig)	11220	11122	16821

Number of genomic CDS covered by reliable hits (>0.7 length and > 0.7 identity for CDS)	6178	5819	7160
Fraction of mapped transcriptome contigs	0.308	0.199	0.124
Fraction of reliably mapped transcriptome contigs	0.098	0.026	0.016

Table S3 Comparison of the transcription assemblies versus the bowhead whale (Alaska) transcriptome assembly

	Gray whale	Bowhead whale transcriptome (Greenland)
Number of mapped contigs	94863	382966
Number of target transcriptome contigs with hits	121025	294747
Number of reliably mapped contigs (>0.7 length and > 0.7 identity for a query contig)	75055	195383
Number of target contigs with reliable hits (>0.7 length and > 0.7 identity for the target contig)	79288	155220
Fraction of mapped transcriptome contigs	0.830	0.362
Fraction of reliably mapped contigs	0.657	0.184

References

1. Keane M, Semeiks J, Webb AE, Li YI, Quesada V, Craig T, Madsen LB, van Dam S, Brawand D, Marques PI, Michalak P, Kang L, Bhak J, Yim HS, Grishin NV, Nielsen NH, Heide-Jorgensen MP, Oziolor EM, Matson CW, Church GM, Stuart GW, Patton JC, George JC, Suydam R, Larsen K, Lopez-Otin C, O'Connell MJ, Bickham JW, Thomsen B, de Magalhaes JP. Insights into the evolution of longevity from the bowhead whale genome. Cell Rep. 2015;10:112-22.
2. Seim I, Ma S, Zhou X, Gerashchenko MV, Lee SG, Suydam R, George JC, Bickham JW, Gladyshev VN. The transcriptome of the bowhead whale *Balaena mysticetus* reveals adaptations of the longest-lived mammal. Aging (Albany NY). 2014;6:879-99.
3. Yim HS, Cho YS, Guang X, Kang SG, Jeong JY, Cha SS, Oh HM, Lee JH, Yang EC, Kwon KK, Kim YJ, Kim TW, Kim W, Jeon JH, Kim SJ, Choi DH, Jho S, Kim HM, Ko J, Kim H, Shin YA, Jung HJ, Zheng Y, Wang Z, Chen Y, Chen M, Jiang A, Li E, Zhang S, Hou H, Kim TH, Yu L, Liu S, Ahn K, Cooper J, Park SG, Hong CP, Jin W, Kim HS,

Park C, Lee K, Chun S, Morin PA, O'Brien SJ, Lee H, Kimura J, Moon DY, Manica A, Edwards J, Kim BC, Kim S, Wang J, Bhak J, Lee HS, Lee JH. Minke whale genome and aquatic adaptation in cetaceans. Nat Genet. 2014;46:88-92.
