# Supplementary material for: De novo assembling and primary analysis of genome and transcriptome of gray whale Eschrichtius robustus
Source: BMC Evol Biol. 2017 Dec 28;17(Suppl 2):258. doi: 10.1186/s12862-017-1103-z (PMC5751776; doi:10.1186/s12862-017-1103-z)
Supplement: Supplementary file 10 — Transcriptome read mapping statistics. See Additional file 7 for the data sources overview. (PDF 12 kb) [file 12862_2017_1103_MOESM10_ESM.pdf]

Transcriptome read mapping statistics

Specimen ID	Total read count after trimming	Mapped reads (unpaired mode)	Mapped reads (paired mode)	Correctly paired reads
bowhead_kidney1	70748234	55691708	55622994	38670946
bowhead_kidney2	76299640	58439682	60202648	42073914
bowhead_kidney3	58651082	45739322	46278862	33147354
bowhead_kidney4	63716658	46115982	50420498	32504022
bowhead_liver1	56513824	46548106	46437126	36214272
bowhead_liver2	68031334	56287658	55878662	43822036
bowhead_liver3	67000946	54045924	54161676	41020994
gray_kidney_mother1	32928788	30523534	27017448	23495184
gray_liver1	52344570	48209046	43384260	36975004
minke_ kidney1	69680452	51735856	56264316	38087696
minke_ liver1	57539188	48399998	46758994	37358692

See Additional file 7 for the data sources overview.
