# Supplementary material for: De novo assembling and primary analysis of genome and transcriptome of gray whale Eschrichtius robustus
Source: BMC Evol Biol. 2017 Dec 28;17(Suppl 2):258. doi: 10.1186/s12862-017-1103-z (PMC5751776; doi:10.1186/s12862-017-1103-z)
Supplement: Supplementary file 12 — Differential gene expression. (PDF 377 kb) [file 12862_2017_1103_MOESM12_ESM.pdf]

Differential gene expression

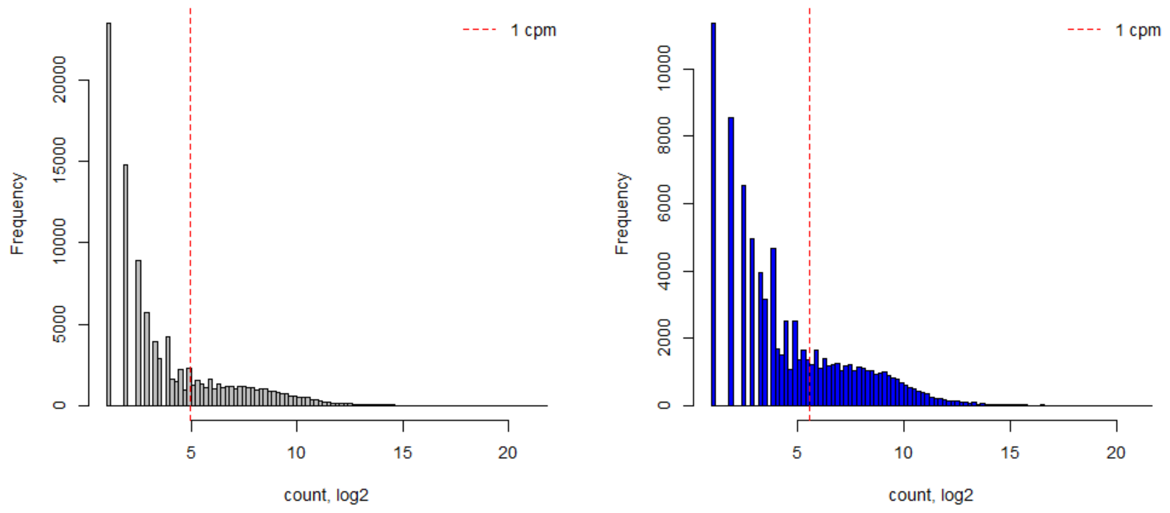

Mapped read counts at contigs with ORFs for kidney (left panel) and liver (right panel). X Axis: log2 read count, Y Axis: number of contigs with a given number of mapped reads. The red dashed line denotes the 1cpm threshold.

The data was normalized using standard edgeR TMM method, the 4th replicate of the bowhead kidney sample [1] was excluded due to abnormal value of the normalization factor.

Since the bowhead whale data was the only for which there were several replicates, it was used as a basis for the comparison (to compute FDR and FoldChange). The following rules were defined to select genes specific for the gray whale transcriptome in comparison to bowhead and minke whale data:

(a) activation (upregulation): FDR < 0.05, log2 FoldChange ≥ 1 or repression (downregulation): FDR < 0.05, log2 FoldChange ≤ -1.

(b) a particular gene is not found as differentially expressed (upregulated or downregulated, respectively) in minke whale transcriptome in comparison to the bowhead whale.

	Kidney (annotated contigs)	Kidney (all contigs)	Liver (annotated contigs)	Liver (all contigs)
Number of contigs passing 1cpm threshold	22570	31257	20187	28628
Upregulated (Gray vs bowhead whale)	807	1993	2579	4951
Upregulated (Gray vs bowhead whale, excluding those detected for minke vs bowhead whale)	271	809	1424	2697

Downregulated (Gray vs bowhead whale)	338	435	2596	3412
Downregulated (Gray vs bowhead whale, excluding those detected for minke vs bowhead whale)	79	107	1264	1601

The resulting absolute number of downregulated genes is quite small which is consistent with the fact that the 1cpm threshold was used for the gray whale data only, and the gray transcriptome assembly is used as the reference thus the lowly expressed transcripts are likely to be completely non-assembled and not present in the mapping reference. Another important observation is that the notable fraction of differentially expressed contigs does not carry annotated CDSs. This means the annotation indeed allows to significantly reduce the number of false positive contigs in the transcriptome assembly.

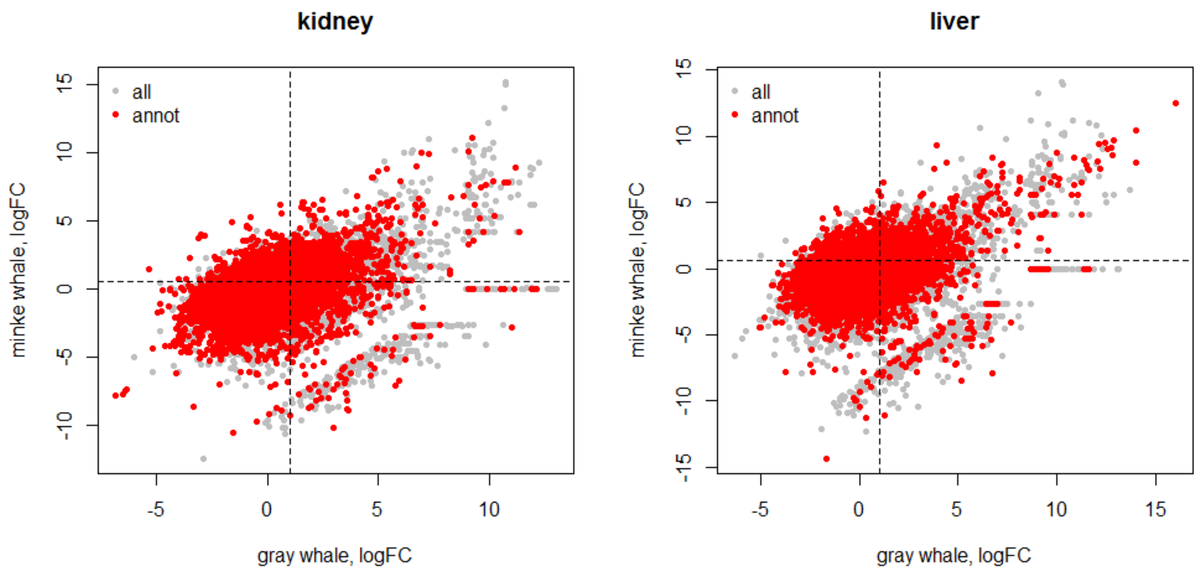

Graphical comparison of log2 FoldChange (vs bowhead whale data) values for gray whale (X axis) and minke whale (Y axis) shows a clear set of non-reliable contigs lying on the linear trend in the bottom-right part of the graph. These contigs are specific for the gray whale transcriptome and basically have only few mapped reads in other transcriptomes (positive log2 FC versus the bowhead whale; negative values of minke-vs-bowhead log2 FC), since the 1cpm threshold was used for gray whale transcriptome data only. Gray points: non-annotated contigs, red points: annotated contigs. Dashed lines correspond to the 2x positive Fold Change (vertical line, for the gray whale) and 1.5x positive Fold Change (horizontal line, for the minke whale).

There is no clear pattern of differential expression, and consideration of specific predefined gene groups, which are assumed to be reliably expressed, also supports this observation.

We have tested genes of the DNA repair (124 annotated contigs of 129 genes presented in MacRae et al. [2] and hypoxia-response systems (ARNT, EPAS1, ARNT2, BMAL1, HIF1N, VHL, NRF1, NF2L2, PRGC1, AAPK2, SIR1, ANGP4) which were both found stably expressed in all studies transcriptomes.

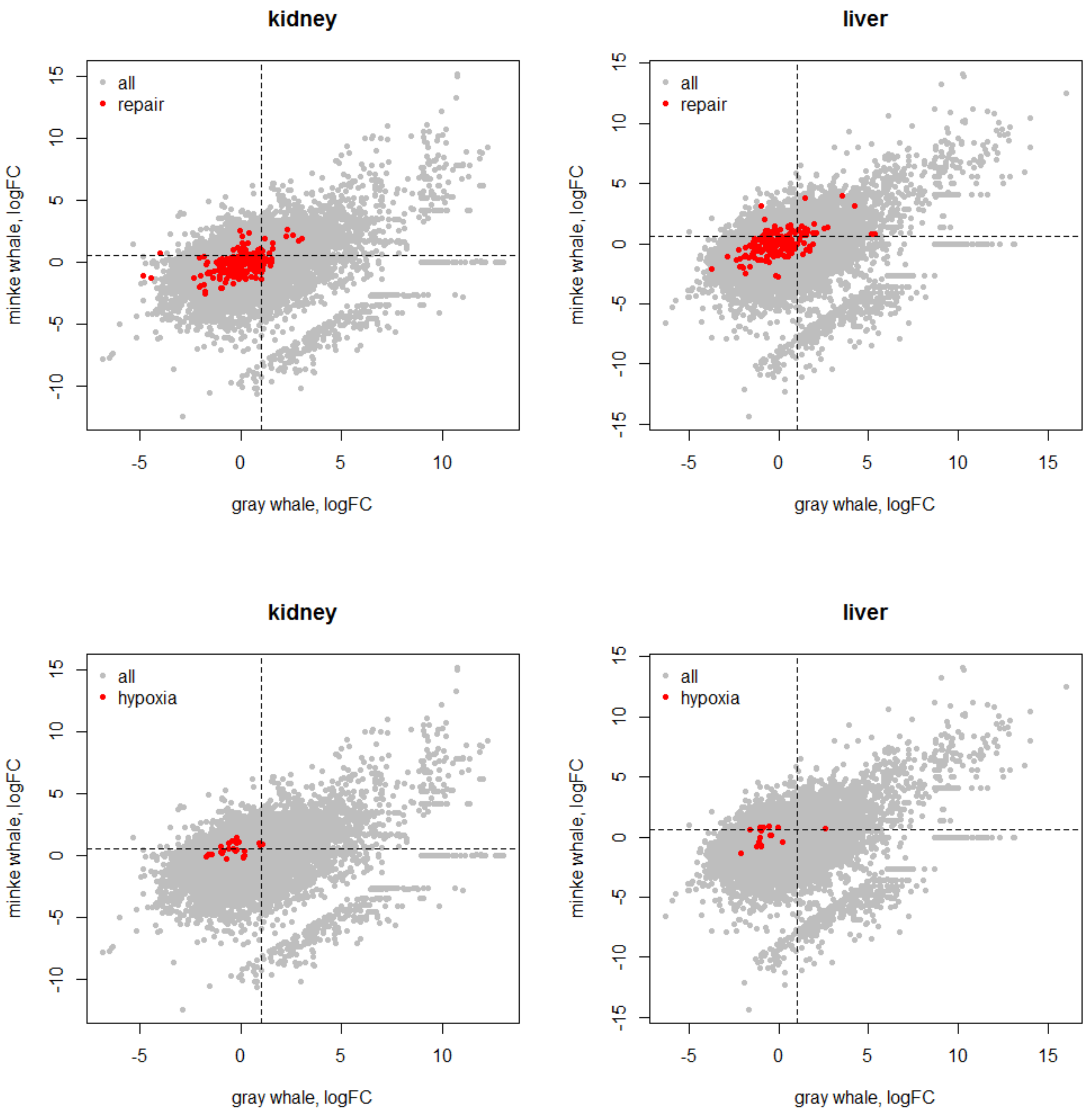

Top panel: DNA repair genes, bottom panel: hypoxia-response genes.

References

1. Seim I, Ma S, Zhou X, Gerashchenko MV, Lee SG, Suydam R, George JC, Bickham JW, Gladyshev VN. The transcriptome of the bowhead whale *Balaena mysticetus* reveals adaptations of the longest-lived mammal. Aging (Albany NY). 2014;6:879-99.
2. MacRae SL, Croken MM, Calder RB, Aliper A, Milholland B, White RR, Zhavoronkov A, Gladyshev VN, Seluanov A, Gorbunova V, Zhang ZD, Vijg J. DNA repair in species with extreme lifespan differences. Aging (Albany NY). 2015;7:1171-84.
